# One-year natural course of corneal densitometry in high myopic patients after implantation of an implantable collamer lens (model V4c)

**DOI:** 10.1186/s12886-020-1320-x

**Published:** 2020-02-12

**Authors:** Xun Chen, Yang Shen, Haipeng Xu, Xiaoying Wang, Xingtao Zhou

**Affiliations:** 1 The Eye and ENT Hospital of Fudan University, 19 Baoqing Road, Xuhui District, Shanghai, Zip code: 200031 China; 2 NHC Key Lab of Myopia (Fudan University), Shanghai, China; 3 Shanghai Research Center of Ophthalmology and Optometry, Shanghai, China

**Keywords:** Implantable collamer lens, Corneal densitometry, Endothelial cell density

## Abstract

**Background:**

Corneal densitometry, which is also known as corneal backscattering, is a surrogate measure of corneal clarity. The purpose of the study was to investigate the changes in corneal densitometry (CD) after implanting an implantable collamer lens (ICL-V4c).

**Method:**

Twenty-six high myopic patients (aged 29.3 ± 6.6 years, 6 males and 20 females) who underwent ICL-V4c implantation were enrolled. Intraocular pressure (IOP), corneal topography, corneal densitometry, uncorrected distance visual acuity (UCDVA), manifest refraction, and best corrected distance visual acuity (BCDVA) were evaluated pre-operatively and at 1 day, 1 week, and 1, 3, 6, and 12 months post-operatively. Endothelial cell density (ECD) was measured pre-operatively and at 3, 6, and 12 months post-operatively. The efficacy index (mean post-operative UCDVA / mean pre-operative BCDVA) and the safety index (mean post-operative BCDVA / mean pre-operative BCDVA) were evaluated at 1 month, 3 months, 6 months and 12 months post-operatively.

**Results:**

Over the annular diameters of 0–2 mm, the pre-operative densitometry values of the anterior layer, central layer, posterior layer, and total layer were 20.1 ± 2.8, 11.8 ± 1.1, 10.5 ± 0.9 and 14.1 ± 1.5, respectively. From pre-operatively to post-operative Month 12, the values changed insignificantly (*P* = 0.177, *P* = 0.153, *P* = 0.543 and *P* = 0.207, respectively). Over the annular diameters of 2–6 mm, the pre-operative mean densitometry values were 17.9 ± 2.2, 10.5 ± 0.9, and 12.6 ± 1.2, respectively. From pre-operatively to post-operative Month 12, the values decreased to 16.5 ± 2.1, 10.0 ± 0.9, and 11.9 ± 1.2, respectively, which were similar to the pre-operative values (all *P* > 0.05) but significantly lower than the values obtained at post-operative Day 1 (*P* = 0.013, *P* = 0.002 and *P* = 0.010, respectively). The densitometry value of the posterior layer over the annular diameters of 2 to 6 mm remained unchanged (from 9.4 ± 0.7 to 9.1 ± 0.7) over time (*P* = 0.372). The efficacy and safety indices assessed at 12 months post-operatively were 1.04 ± 0.27 and 1.19 ± 0.23, respectively. The changes in IOP and ECD values were statistically insignificant (*P* = 0.896 and *P* = 0.968, respectively).

**Conclusion:**

ICL-V4c implantation may be safe and efficient for high ametropia correction. The corneal densitometry values obtained over the annulus of 0–6 mm increased slightly from before the operation to post-operative Day 1 and then decreased gradually, which indicates that ICL-V4c implantation may not compromise corneal clarity.

## Background

Corneal densitometry (also known as corneal backscatter) is an objective method for evaluating corneal clarity and transparency [1–4]. Studies have reported that corneal diseases such as keratitis [5], corneal epithelial oedema [6], Fuchs’ endothelial corneal dystrophy [7] and keratoconus [8] can compromise corneal clarity, thus leading to an increase in corneal densitometry. The normal function of corneal endothelium and the regular arrangement of stromal layers play important roles in maintaining corneal clarity [9]. Otri AM [10] has noted that corneal densitometry can be used as an indicator of corneal heath.

The implantation of a posterior chamber implantable collamer lens (ICL) has been considered an effective procedure for myopia correction [11–13]. Since ICL implantation avoids ablating or removing corneal tissue, the integrity of the corneal structure and the stability of the corneal biomechanics are theoretically maintained; hence, ICL implantation, compared with corneal refractive surgeries, may minimize the risk of ectasia [14–19]. However, complications such as endothelial cell damage and intraocular pressure (IOP) elevation have also been reported [20] intraoperatively or post-operatively.

ICL model V4c (ICL-V4c, STAAR Surgical Company, Monrovia, California, USA) is a modified posterior chamber ICL, which is designed with a central hole instead of iridectomy to improve aqueous humour circulation. Studies have reported that ICL-V4c and conventional ICLs (without a central hole) present similar visual outcomes [21–24]. Nevertheless, surgery-associated endothelial damage, including endothelial cell trauma and endothelial oedema, may not be completely avoided [25]. Moreover, the potential intraoperative or post-operative ocular hypertension (OHT) and IOL rise may also compromise the corneal endothelial barrier and may lead to corneal oedema, affecting corneal transparency [26]. The purpose of this study was to investigate the natural course of corneal densitometry after ICL-V4c implantation.

## Methods

### Participants

Twenty-six high myopic patients (6 males and 20 females) who had not yet received the ICL-V4c operation were selected for this study. Each patient had the operation on only 1 eye. The mean age of the patients was 29.3 ± 6.6 years (range 18 to 44 years). The mean value of manifest refraction spherical equivalent (MRSE) was − 12.48 ± 3.79 dioptres (D) (range − 26.63 to − 6.63 D).

The inclusion criteria were the following: age ≥ 18 years, MRSE of higher than − 6.00 D, astigmatism of up to − 5.00 D, best corrected distance visual acuity (BCDVA) of 20/40 or better, anterior chamber depth of ≥2.8 mm, and endothelial cell density of ≥2000 cell/mm2.

The exclusion criteria were the following: a history of suspected keratectasia, cornea or lens opacity, retinal detachment, glaucoma, macular degeneration, or neuro-ophthalmic disease, a history of ocular surgery, inflammation or trauma, systemic disease, and pregnancy.

Ethics approval and consent to participate were obtained. This study adhered to the Declaration of Helsinki and was approved by the Ethical Committee Review Board of Fudan University Eye and ENT Hospital. Written informed consent was obtained from each patient after the procedure was thoroughly explained.

### Ophthalmic examinations

Each patient underwent routine pre-operative evaluations. Intraocular pressure, corneal topography and corneal densitometry were assessed using a non-contact tonometer and an anterior segment analyser (Pentacam HR, Typy70900, Oculus Optikgeräte GmbH, Germany), respectively. The evaluations were administered before the operation and at 1 day, 1 week, 1 month, 3 months, 6 months and 12 months after the operation.

Corneal densitometry values measured from the Scheimpflug images are expressed in grey scale units (range 0 to 100) and are presented in the Average Cornea Densitometry Table (version 1.20r29). The mean densitometry values on the diameters from 0 to 2 mm and 2–6 mm of the corneas’ anterior layer (the first 120 μm of the complete corneal thickness), posterior layer (the last 60 μm of the complete corneal thickness), central layer (the volume between the anterior layer and the posterior layer), and the total layer (the volume between the epithelium and endothelium of a cornea) were analysed since densitometry measurements were much more accurate and repeatable in the central areas than in the peripheral areas [27, 28].

The efficacy index (mean post-operative UCDVA / mean pre-operative BCDVA) and the safety index (mean post-operative BCDVA / mean pre-operative BCDVA) were also evaluated at 12 months post-operatively. UCDVA, manifest refraction, BCDVA, and IOP (non-contact tonometer) were all assessed before the operation and at 1 month, 3 months, 6 months and 12 months after the operation. Corneal endothelial cell density (ECD) was measured with a specular microscope (SP-2000P; Topcon Corporation, Japan) before the operation and at 6 months and 12 months after the operation.

### Surgical techniques

All ICL-V4c procedures were performed by the same surgeon (XYW) under cycloplegia (2.5% phenylephrine and 1% tropicamide, Alcon, China) and topical anaesthesia (0.4% oxybuprocaine hydrochloride, Santen, Japan). A lid speculum was used to fully expose the ocular surface after the sterilization of the ocular surface and periocular skin (povidone-iodine 5%). Then, a 3 mm incision was created at the temporal or superior corneoscleral limbus. After a viscoelastic surgical agent (1.7% sodium hyaluronate; Bausch & Lomb, China) was injected into the anterior chamber to maintain the anterior chamber depth, an ICL-V4c was inserted into the anterior chamber through the incision with an injector cartridge. An additional viscoelastic agent was then placed on the top of the ICL (between the corneal endothelium and the upper surface of the ICL-V4c), and an ICL positioning instrument was used to sweep the 4 footplates of the ICL beneath the iris. Subsequently, both the anterior chamber and the posterior chamber were irrigated thoroughly using a balanced salt solution. Finally, the overfilled perfusate was released from the incision, and the IOP was estimated with “finger touch” before the end of the procedure.

### Post-operative topical therapies

Each patient was required to use antibiotic eyedrops (0.5% left ofloxacin, Santen, Japan), steroidal eye drops (prednisolone acetate ophthalmic suspension 1%, Allergan Pharmaceuticals, Ireland), and miotic eye drops (pilocarpine nitrate 0.5%, Bausch & Lomb, USA) 4 times every 10 min immediately after the procedure. Antibacterial and steroidal medications (0.1% tobramycin dexamethasone, Alcon, China) were prescribed to be taken 4 times daily for 3 days, followed by fluorometholone eye drops that were tapered gradually over 2 weeks. Then, antibiotic eyedrops (0.5% left ofloxacin, Santen, Japan) were prescribed to be taken 4 times daily for 1 week, non-steroidal anti-inflammatory eyedrops (NSAID, pranoprofen, Senju, Japan) were prescribed to be taken 4 times daily for 2 weeks and artificial tears were prescribed to be taken 4 times daily for 1 month [29].

### Data analysis and statistical evaluation

All the values are expressed as the mean ± standard deviation. Statistical analyses were performed using SPSS 19 (SPSS Inc., IBM). A normality check was conducted using the Kolmogorov–Smirnov test. A mixed linear model with Bonferroni-adjusted post hoc comparisons was used to evaluate the changes in MRSE, CD, ECD and IO*P* values over time. The cut-off *P* value was 0.05 for indicating statistical significance.

## Results

All procedures were completed without complications. At 2 h after ICL-V4c implantation, each post-operative eye had mild epithelial oedema and endothelial oedema, which were identified with slit lamp biomicroscopy. However, the cornea recovered the day after the operation. No severe intraoperative or post-operative complications occurred.

### The natural course of corneal densitometry after ICL implantation

**Fig. 1:**
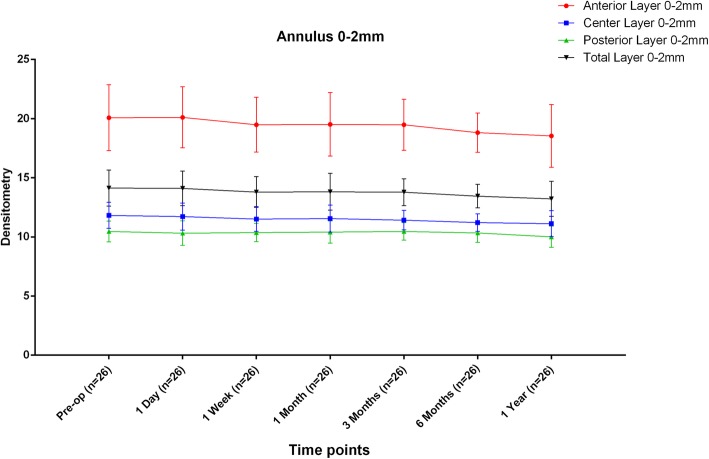
Densitometry over the annular diameters 0–2 mm of the cornea at different follow-up times after ICL V4c implantation

The mean densitometry values obtained before and after ICL implantation are listed in Table 1. Over the annular diameters of 0–2 mm, no significant changes were detected in the densitometry values of the anterior layer (AL 0–2 mm), the central layer (CL 0–2 mm), the posterior layer (PL 0–2 mm) or the total layer (TL 0–2 mm) (*F* = 1.510, *P* = 0.177; *F* = 1.589, *P* = 0.153; *F* = 0.836, *P* = 0.543 and *F* = 1.426, *P* = 0.207, respectively) within the first year after the operation (Fig. 1).

**Table 1:** The natural course of corneal densitometry (*n* = 26)

Variables	Pre-op	1 Day	1 Week	1 Month	3 Months	6 Months	1 Year	*F* value^a^	*P* value
		Post-op	Post-op	Post-op	Post-op	Post-op	Post-op		
	Mean±SD	Mean±SD	Mean±SD	Mean±SD	Mean±SD	Mean±SD	Mean±SD		
AL0-2 mm	20.1 ± 2.8	20.1 ± 2.6	19.5 ± 2.3	19.5 ± 2.7	19.5 ± 2.2	18.8 ± 1.7	18.5 ± 2.7	1.510	0.177
CL0-2 mm	11.8 ± 1.1	11.7 ± 1.1	11.5 ± 1.1	11.5 ± 1.1	11.4 ± 0.8	11.2 ± 0.7	11.1 ± 1.1	1.589	0.153
PL0-2 mm	10.5 ± 0.9	10.3 ± 1.0	10.4 ± 0.8	10.4 ± 0.9	10.5 ± 0.7	10.3 ± 0.8	10.0 ± 0.9	0.836	0.543
TL0-2 mm	14.1 ± 1.5	14.1 ± 1.5	13.8 ± 1.3	13.8 ± 1.5	13.8 ± 1.1	13.5 ± 1.0	13.2 ± 1.5	1.426	0.207
AL2-6 mm	17.9 ± 2.2	18.5 ± 2.2	17.5 ± 2.0	17.3 ± 2.3	17.2 ± 1.8	16.7 ± 1.4^b^	16.5 ± 2.1^b^	2.798	0.013^*^
CL2-6 mm	10.5 ± 0.9	11.0 ± 1.1	10.3 ± 0.9	10.3 ± 1.0	10.2 ± 0.8^b^	10.0 ± 0.7^b^	10.0 ± 0.9^b^	3.750	0.002^**^
PL2-6 mm	9.4 ± 0.7	9.5 ± 0.9	9.4 ± 0.7	9.4 ± 0.8	9.4 ± 0.7	9.3 ± 0.7	9.1 ± 0.7	1.087	0.372
TL2-6 mm	12.6 ± 1.2	13.0 ± 1.3	12.4 ± 1.2	12.3 ± 1.4	12.3 ± 1.0	12.0 ± 0.9	11.9 ± 1.2^b^	2.719	0.015^*^

*Pre-op* Pre-operation, *Post-op* Post-operation, *AL* Anterior Layer, *CL* Central Layer, *PL* Posterior Layer, *TL* Total Layer

* *P*<0.05; ** *P*<0.01

^a^ Mixed linear model

^b^ significant difference was detected when compared with the value obtained at the 1 day mark

**Fig. 2:**
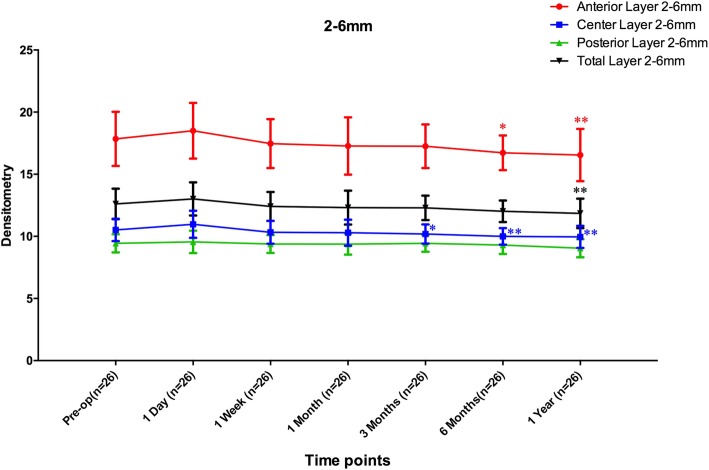
Densitometry over the annular diameters 2–6 mm of the cornea at different follow-up times after ICL V4c implantation. (* refers to the *P* value < 0.05; ** refers to the *P* value < 0.01)

Over the annular diameters of 2–6 mm, the mean densitometry values of the anterior layer (AL 2–6 mm), the central layer (CL 2–6 mm), and the total layer (TL 2–6 mm) changed significantly (*F* = 2.798, *P* = 0.013; *F* = 3.750, *P* = 0.002 and *F* = 2.719, *P* = 0.015, respectively). Bonferroni-adjusted post hoc comparisons revealed that all the post-operative densitometry values obtained from the four layers were similar to the values measured pre-operatively (all *P* values > 0.05). However, the densitometry values of the AL 2–6 mm obtained at 6 months and 12 months after the operation were significantly lower than the value obtained at 1 day after the operation (*P* = 0.039 and *P* = 0.013). The values of CL 2–6 mm assessed at 3 months, 6 months and 12 months after the operation significantly decreased compared with the value obtained at post-operative Day 1 (*P* = 0.046; *P* = 0.004 and *P* = 0.002, respectively). The densitometry values of TL 2–6 mm obtained at post-operative Month 12 were significantly lower than those obtained at 1 day after the operation (*P* = 0.010) (Fig. 2).

### Visual outcomes

**Fig. 3:**
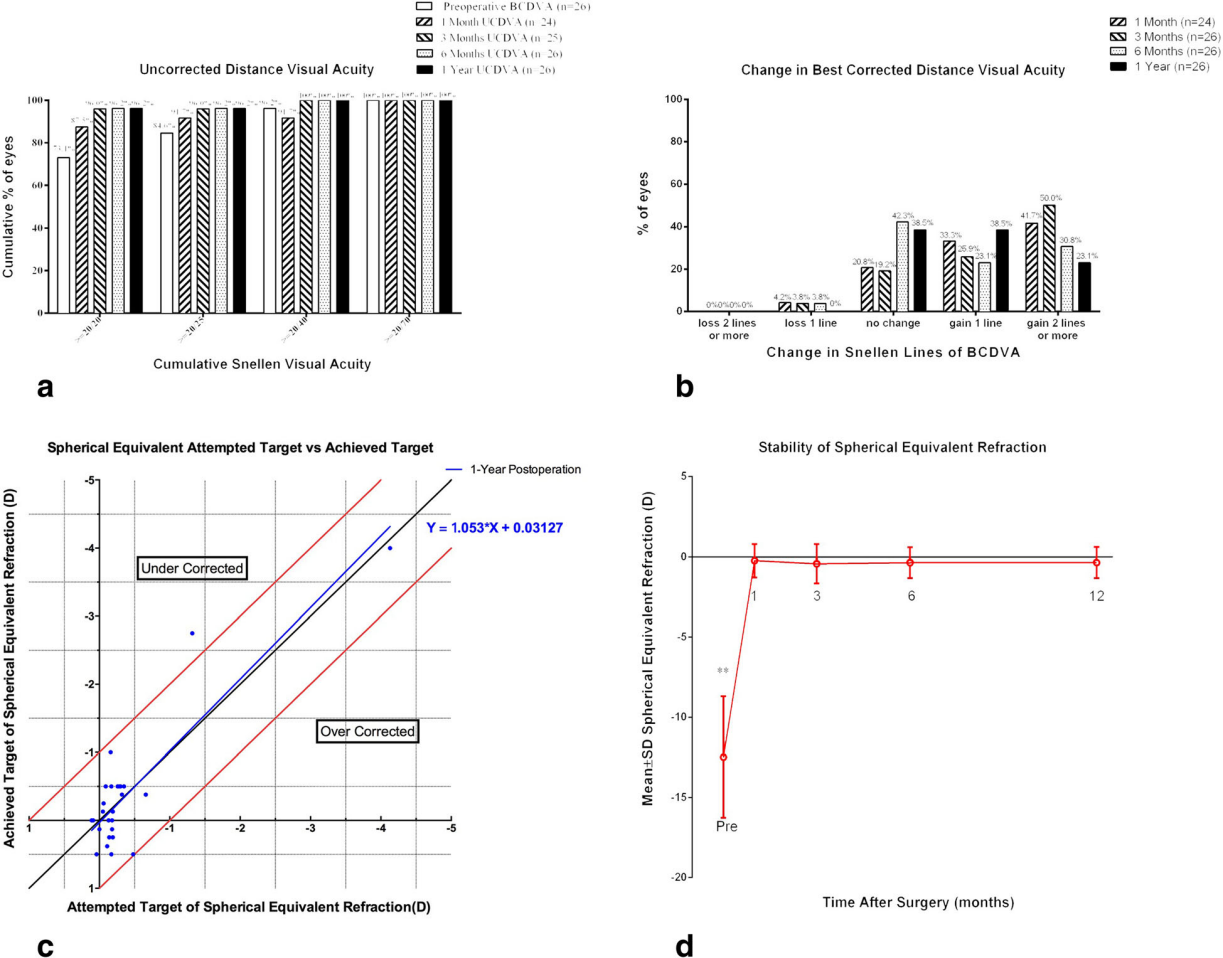
Refractive outcomes (**a**: Efficacy **b**: Safety **c**: Predictability **d**: Stability) at different follow-up times after the implantation of an implantable collamer lens with a central hole (ICL V4c). UCDVA = uncorrected distance visual acuity; BCDVA = best corrected distance visual acuity; D = dioptres; SD = standard deviation

As demonstrated in Fig. 3a to d, 19 eyes (73.1%) had a UCDVA of 20/20 or better, 22 eyes (84.6%) had a UCDVA of 20/25 or better, 25 eyes (96.2%) had a UCDVA of 20/40 or better, and all 26 eyes (100%) had a UCDVA of 20/70 or better (Fig. 3a). No statistically significant difference was detected between the BCDVA before the operation and UCDVA (*F* = 1.032, *P* = 0.394) after the operation. The efficacy indices, assessed at 1 month, 3 months, 6 months and 12 months, were 1.13 ± 0.32 (*n* = 24), 1.13 ± 0.24 (*n* = 26), 1.09 ± 0.25 (*n* = 26) and 1.04 ± 0.27 (*n* = 26), respectively. At the 12-month mark, no patient showed a decline in BCDVA; 10 eyes (38.5%) had unchanged BCDVA, another 10 eyes gained one line (38.5%), and the remaining 6 eyes (23.1%) gained 2 or more lines (Fig. 3b). The safety indices assessed at the 1-month, 3-month, 6-month and 12-month marks were 1.27 ± 0.26 (*n* = 24), 1.29 ± 0.23 (*n* = 26), 1.19 ± 0.26 (*n* = 26), and 1.19 ± 0.23 (*n* = 26), respectively. A scatterplot and the best linear fit line of the attempted versus the achieved spherical equivalent refraction (SER) correction are shown in Fig. 3c. The values of manifest spherical equivalent refraction (MSER) measured before the operation and at 1 month, 3 months, 6 months, and 12 months after were − 12.48 ± 3.79 D, − 0.24 ± 1.04 D, − 0.44 ± 1.23 D, − 0.37 ± 0.97 D and − 0.36 ± 0.98 D, respectively. As shown in Fig. 3d, the SER before the operation was significantly lower than those after the operation (all *P* values < 0.001). However, there were no significant differences among the SER values after the operation (all *P* values > 0.05).

### IOP and ECD outcomes

The mean IOP values measured before the procedure and at the 1-day, 1-week, 1-month, 3-month, 6-month and 12-month marks were 15.43 ± 3.00 mmHg, 14.58 ± 3.76 mmHg, 15.27 ± 3.77 mmHg, 14.90 ± 3.46 mmHg, 15.48 ± 3.84 mmHg, 14.55 ± 3.78 and 15.52 ± 2.87 mmHg, respectively. The mean ECD values before the procedure and at 3 months, 6 months and 12 months after were 3273.9 ± 342.9/mm2, 3263.9 ± 437.6/mm2, 3311.7 ± 459.2/mm2 and 3261.4 ± 355.1/mm2, respectively. The mean IOP (F = 0.372, *P* = 0.896) and ECD (F = 0.085, *P* = 0.968) values remained unchanged over time.

## Discussion

ICL procedures correct refractive errors without removing corneal tissue, thus avoiding the risk of secondary corneal ectasia. Currently, ICL procedures are becoming increasingly popular, especially for high ametropia correction [13, 14].

The results of the current study demonstrated that the densitometry values of AL 0–2 mm, CL 0–2 mm, PL 0–2 mm and TL 0–2 mm remained unchanged during the 12-month follow-up period, which was consistent with the ECD results. In addition, the values of AL 2–6 mm, CL 2–6 mm, PL 2–6 mm and TL 2–6 mm measured at 1 day, 1 month, 3 months, 6 months and 12 months after the procedure were also unchanged compared with those measured before the procedure, indicating that the effects of ICL-V4c implantation on central corneal densitometry may be insignificant and that the corneal histological structure is intact. The mean values of AL 2–6 mm, CL 2–6 mm and TL 2–6 mm increased slightly at the 1-day mark but then decreased gradually until post-operative Month 12. Significant differences in the values of AL 2–6 mm, CL 2–6 mm and TL 2–6 mm were detected between post-operative Day 1 and post-operative Month 12. We hypothesize that the surgical operation may slightly compromise the transparency of the peripheral cornea. Dong et al. [30] investigated the differences in corneal densitometry between high myopic patients and normal individuals and found that densitometry values of the high myopic patients were significantly lower than those of the normal individuals. The authors hypothesize that the natural loss of endothelial cell density in a high myopic population might lead to a decrease in corneal densitometry. However, in the present study, although we detected a significant decrease in corneal densitometry between post-operative Day 1 and post-operative Month 12, we did not detect any remarkable changes in endothelial cell density. We suggest that changes in corneal densitometry may be caused by various factors, including the density and function of endothelial cells, the balance between the stromal swelling pressure and the endothelial pump rate [31, 32], the corneal wound healing reaction and even the quality of tear film [33]. Additional studies are needed to determine the primary cause of a decrease in corneal densitometry.

Ní Dhubhghaill S et al. [27] investigated normative corneal densitometry data in a normal population. The authors found that the densitometry value is the lowest in the central zone and the highest in the anterior layer, which is consistent with our results. However, Ní Dhubhghaill S’s data showed that the accurate corneal densitometry values of each zone and each layer were much higher than our corresponding values. We suggest that racial difference may also be a major factor that affects corneal densitometry values, as Caucasians’ corneas are thicker than Asians’ corneas [34].

Pentacam HR was able to assess the corneal densitometry values over the annular zones of 0–12 mm; however, as most Asians’ palpebral fissures are narrow, their eyelids and eyelashes cover the peripheral areas of annuli 6–10 mm and 10–12 mm; thus, we have to analyse the zones over the annuli of 0–2 mm and 2–6 mm instead of the entire cornea. This method of measurement is a limitation of the study.

In this study, we found that at post-operative Month 12, both the MSER and visual acuity (VA) remained stable. The safety and efficacy indices were 1.19 ± 0.23 and 1.04 ± 0.27, there were no declines in BCDVA, and all the patients were satisfied with their vision, implying that ICL-V4c implantation is safe and efficient for high ametropia correction. Pérez-Vives C [35] and Ganesh S [36] investigated the differences in visual and refractive results between the ICL procedure and corneal refractive surgery. The outcomes demonstrated that the quality of vision and patient satisfaction is better with ICL than with femtosecond-LASIK. Tian Y [37] and Hyun J [38] compared the differences in visual and refractive results between ICL-V4 and ICL-V4c. Both authors found that the central hole did not significantly affect the visual and refractive outcomes. However, Eppig T et al. analysed the optical effect of the central hole on the ICL. The authors found that all the eye models showed ghost images and noted that the central hole may cause stray light rays and ghost images; however, the on-axis visual quality was mostly unaffected [39]. Additional studies are required to determine the effects of the central hole on visual quality and patient satisfaction.

Ocular hypertension (OHT) is considered one of the most commonly observed symptoms after ICL implantation. The possible causes include steroid responses, retained viscoelastic agents, acute pupillary blocks, and pigment dispersion [26]. In the current study, the IOP values changed slightly over time. The possible reasons may be as follows.

The short-term usage of steroidal eye drops and the application of an NSAID may maximally avoid steroid-related ocular hypertension, which was reported as the most common cause of OHT after ICL implantation.

As the central hole joins the anterior chamber and the posterior chamber, laser peripheral iridotomy is no longer routinely recommended before ICL-V4c implantation. Hence, the risks of pigment dispersion and acute pupillary block may be remarkably reduced. Thorough irrigation of the anterior and posterior chambers may be another key step that prevents retained viscoelastic agent-related OHT.

During the ICL-V4c implantation procedure, the intraocular space for surgical operation is limited, and endothelial cell damage (including endothelial cell loss or endothelial oedema) is a potential complication, especially when an ICL is being injected into the anterior chamber or positioned into the posterior chamber [20, 40, 41]. In the current study, we monitored the ECD values for 12 months and found that the mean ECD values measured pre-operatively and at 3 months, 6 months and 12 months post-operatively were indistinctive, implying that ECD may have been unaffected after ICL-V4c implantation. Therefore, ICL-V4c implantation may be safe for endothelial cells.

However, because the specular microscope evaluates ECD by analysing the central corneal image, which was taken through a central fixation area of the cornea [42, 43], peripheral endothelial damage may not be detected. Corneal densitometry assessments by using a Pentacam HR are able to detect various abnormalities in a wider zone than is a specular microscope. Corneal densitometry analysis may be employed to assess endothelial function in peripheral zones [2, 4, 9, 10].

## Conclusions

In summary, ICL-V4c implantation may be safe and efficient for high ametropia correction. The corneal densitometry values obtained over the annulus of 0–6 mm increase slightly at post-operative Day 1 and then decrease gradually, indicating that ICL-V4c implantation may not compromise corneal clarity.

## Data Availability

Data and materials are available upon request from the corresponding author at doctxiaoyingwang@163.com.

## Abbreviations

AL: Anterior layer
BCDVA: Best corrected distance visual acuity
CD: Corneal densitometry
CL: Central layer
D: Dioptres
ECD: Endothelial cell density
ICL: Implantable collamer lens
IOP: Intraocular pressure
MRSE: Manifest refraction spherical equivalent
NSAID: Nonsteroidal anti-inflammatory drug
OHT: Ocular hypertension
PL: Posterior layer
SER: Spherical equivalent refraction
TL: Total layer
UCDVA: Uncorrected distance visual acuity
VA: Visual acuity
